# Consortium on Vulnerability to Externalizing Disorders and Addictions (cVEDA): A developmental cohort study protocol

**DOI:** 10.1186/s12888-019-2373-3

**Published:** 2020-01-02

**Authors:** Eesha Sharma, Preeti Jacob, Pratima Murthy, Sanjeev Jain, Mathew Varghese, Deepak Jayarajan, Keshav Kumar, Vivek Benegal, Nilakshi Vaidya, Yuning Zhang, Sylvane Desrivieres, Gunter Schumann, Udita Iyengar, Bharath Holla, Meera Purushottam, Amit Chakrabarti, Gwen Sascha Fernandes, Jon Heron, Matthew Hickman, Kamakshi Kartik, Kartik Kalyanram, Madhavi Rangaswamy, Rose Dawn Bharath, Gareth Barker, Dimitri Papadopoulos Orfanos, Chirag Ahuja, Kandavel Thennarasu, Debashish Basu, B. N. Subodh, Rebecca Kuriyan, Sunita Simon Kurpad, Kalyanaraman Kumaran, Ghattu Krishnaveni, Murali Krishna, Rajkumar Lenin Singh, L. Roshan Singh, Mireille Toledano

**Affiliations:** 1 Department of Child and Adolescent Psychiatry, National Institute of Mental Health and Neuro-Sciences (NIMHANS), Bangalore, Karnataka India; 2 Department of Psychiatry, National Institute of Mental Health and Neuro-Sciences (NIMHANS), Bangalore, Karnataka India; 3 Centre for Population Neuroscience and Precision Medicine, Institute of Psychology, Psychiatry & Neuroscience, MRC SGDP Centre, King’s College London, London, UK; 4 Department of Child & Adolescent Psychiatry, Institute of Psychology, Psychiatry & Neuroscience, King’s College London, London, UK; 5 Molecular Genetics Laboratory, National Institute of Mental Health and Neuro-Sciences (NIMHANS), Bangalore, Karnataka India; 6 Regional Occupational Health Centre (ROHC), Eastern, ICMR-National Institute of Occupational Health (NIOH), Kolkata, West Bengal India; 7 Population Health Sciences, Bristol Medical School, University of Bristol, Bristol, UK; 8 Rishi Valley Rural Health Centre, Madanapalle, Chittoor, Andhra Pradesh India; 9 Department of Psychology, Christ University, Bangalore, Karnataka India; 10 Department of Neuroimaging and Interventional Radiology, National Institute of Mental Health and Neuro-Sciences (NIMHANS), Bangalore, Karnataka India; 11 Department of Neuroimaging, Institute of Psychology, Psychiatry & Neuroscience, King’s College London, London, UK; 12 NeuroSpin, CEA, Université Paris-Saclay, Paris, France; 13 Department of Radiodiagnosis and Imaging, Post Graduate Institute of Medical Education and Research (PGIMER), Chandigarh, India; 14 Department of Biostatistics, National Institute of Mental Health and Neuro-Sciences (NIMHANS), Bangalore, Karnataka India; 15 Department of Psychiatry, Post Graduate Institute of Medical Education and Research (PGIMER), Chandigarh, India; 16 Division of Nutrition, St John’s Research Institute, Bengaluru, India; 17 Department of Psychiatry and Department of Medical Ethics, St. John’s Medical College and Hospital, Bengaluru, India; 18 MRC Lifecourse Epidemiology Unit, University of Southampton, Southampton, UK; 19 Epidemiology Research Unit, CSI Holdsworth Memorial Hospital, Mysore, India; 20 Foundation for Research and Advocacy in Mental Health, Mysore, India; 21 Department of Psychiatry, Regional Institute of Medical Sciences (RIMS), Imphal, Manipur India; 22 Department of Clinical Psychology, Regional Institute of Medical Sciences (RIMS), Imphal, Manipur India; 23 Faculty of Medicine, School of Public Health, Imperial College, London, UK

**Keywords:** Externalizing disorders, Study protocol, Vulnerabilities, Longitudinal study, Cohort, Environmental exposures

## Abstract

**Background:**

Low and middle-income countries like India with a large youth population experience a different environment from that of high-income countries. The Consortium on Vulnerability to Externalizing Disorders and Addictions (cVEDA), based in India, aims to examine environmental influences on genomic variations, neurodevelopmental trajectories and vulnerability to psychopathology, with a focus on externalizing disorders.

**Methods:**

cVEDA is a longitudinal cohort study, with planned missingness design for yearly follow-up. Participants have been recruited from multi-site tertiary care mental health settings, local communities, schools and colleges. 10,000 individuals between 6 and 23 years of age, of all genders, representing five geographically, ethnically, and socio-culturally distinct regions in India, and exposures to variations in early life adversity (psychosocial, nutritional, toxic exposures, slum-habitats, socio-political conflicts, urban/rural living, mental illness in the family) have been assessed using age-appropriate instruments to capture socio-demographic information, temperament, environmental exposures, parenting, psychiatric morbidity, and neuropsychological functioning. Blood/saliva and urine samples have been collected for genetic, epigenetic and toxicological (heavy metals, volatile organic compounds) studies. Structural (T1, T2, DTI) and functional (resting state fMRI) MRI brain scans have been performed on approximately 15% of the individuals. All data and biological samples are maintained in a databank and biobank, respectively.

**Discussion:**

The cVEDA has established the largest neurodevelopmental database in India, comparable to global datasets, with detailed environmental characterization. This should permit identification of environmental and genetic vulnerabilities to psychopathology within a developmental framework. Neuroimaging and neuropsychological data from this study are already yielding insights on brain growth and maturation patterns.

## Background

India is home to the world’s largest number of adolescents and young people (10–24 years old), comprising about a third (>400 million) of its population [1]. Nearly 20% of young people experience a mental health condition, bearing a disproportionately high burden of mental morbidity [2–4]. The Consortium on Vulnerability to Externalizing Disorders and Addictions (cVEDA) is a multi-site, international, collaborative, cohort study in India, setup to examine the interactions of environmental exposures, and genomic influences on neurodevelopmental trajectories and downstream vulnerability to psychopathology, with a specific focus on externalizing spectrum disorders. cVEDA spans seven recruitment centres, representing different geographical, physical and socio-cultural environments, across India. This paper presents the background, rationale and protocol of the cVEDA study.

### The need for longitudinal studies using dimensional, multi-modal measures to study the etiopathological basis of psychiatric morbidity

Birth-cohort studies show that >70% psychiatric illnesses in adults begin before the age of 18 years [5]. Temperamental and psychological disturbances during childhood are prominent predictors of psychiatric disorders in adult life [6]. Childhood onset psychiatric disorders often extend into adulthood on a homotypic or heterotypic continuum [7]. Psychiatric morbidity is increasingly believed to be predicated upon a shared genetic and neurodevelopmental continuum, suggested by commonalities in clinical phenotype, neuroimaging characteristics, neuropsychological impairments, and environmental risk factors [8, 9]. Different psychiatric disorders rather than being discrete categories, possibly reflect differences in timing, severity and patterns of genetic expression and neurodevelopmental deviations [10]. Longitudinal studies over the developmental lifespan are ideally suited to study origins of psychiatric morbidity by tracking developmental trajectories, identifying deviations, and studying how deviations, and their interactions with genes and environment, relate to psychopathology [11–13]. Additionally, a combination of neuroimaging, neuropsychological, toxicological, and psychometric modalities better facilitates construction of models with high predictive power [14].

### Role of the ‘exposome’ in determining psychiatric morbidity and the need to study it in low and middle-income countries

Psychiatric disorders have complex, multi-factorial, *poly-gene-environmental* etiologies [15]. Gene x environment correlation (rGE) and interaction (GxE) are two broad mechanisms that underlie this complex interface [16]. The *exposome* [17] includes *general external environment* (socio-economic, habitat), *specific external environment* (pollutants, infectious agents, substance use) and *internal environment* (physical activity, oxidative stress, etc.) [18]. Table 1 depicts the time-dependent impact of environmental exposures on developmental and psychopathological outcomes [19, 20].

**Table 1:** Developmental and psychopathological impact of environmental exposures over different life stages

Life stage	Exposure	Developmental impact	Impact on mental morbidity
Fetus	Maternal mal-nutrition	Early brain development, including key serotonergic and dopaminergic signalling systems	Externalizing problems in early childhood [21]
	Intra-uterine growth retardation/Low birth weight	Developmental programming of physiological systems	Wide range of cognitive, emotional and behavioural outcomes [22]
	Maternal substance use	Later growth and development including trans-generational effects [23]	Wide range of cognitive, emotional and behavioural outcomes
	Psychosocial stress during pregnancy/ maternal depression & anxiety		Behavioural disturbances in later childhood [24]
	Environmental pollutants – toluene in traffic smoke, organophosphates in pesticides	Developmental neurotoxicity; Neuroimaging evidence of structural abnormalities	Cognitive deficits [25, 26]
Early childhood	Pollutants, environmental toxins – polycyclic aromatic hydrocarbons in biomass fuels, tobacco smoke, arsenic in ground water, fluoride, lead, polychlorinated biphenyls from insulators in electrical equipment, phthalates from plastics and cosmetics	Injury to the developing human brain either through direct toxicity or interactions with the genome [27]	Low verbal IQ [29]; Cognitive deficits among preschool-age children [30]; behavioral abnormalities [31]
		Neurotoxicity with effects persistent throughout life [28]	
	Absence of primary attachment figure/poor parenting	Deficits in cognitive and socio-emotional development [32]	Indiscriminate friendliness, poor peer relationships [33]
	Under-nutrition	Synaptic pruning, Myelination, Executive functioning [34, 35]	Risk of emotional and behavioural problems [36]; High prevalence of health-harming behaviours [37]
Childhood & adolescence	Poverty/deprived neighbourhoods	Via parental psychopathology, less positive parenting, neglect, poor monitoring [38]	Higher prevalence of SUDs [39]; various negative behavioural outcomes [40]; Conduct problems [41]
	Exposure to war and conflict		Range of psychopathology, including post-traumatic stress disorder [42]
	High conflict home environment (parental marital conflict, parental divorce)		Disruptive behaviours [43]
	Harsh parenting, physical abuse		Disruptive and emotional psychopathology [44]
	Deviant peer relationships	Behavioural reinforcement, exchange of techniques	Delinquentbehaviours [45]
Adolescence	Substance use	Interferes with brain maturation especially in areas affecting self-regulation and control	Substance use disorders and global difficulties in adult functioning [46]

The exposome, which can potentially modify genetic expression, is multi-cultural. Extrapolating findings from high-income settings to low and middle-income countries (LMIC) is problematic [47]. Ethnic backgrounds, in a diverse country like India [48], and the prevalence of exposures and outcomes pertinent to development and health vary by income group and geography [49–51]. Certain environmental risk factors (nutritional stress, environmental neurotoxins and culturally dependent forms of psychosocial stress) are largely specific to developing societies. Maternal malnutrition, suboptimal breast-feeding, childhood malnutrition, unsafe water, poor sanitation, indoor smoke, and high-risk behaviors are leading causes of death and disability-adjusted life years in LMIC [52, 53]. It is necessary to study the exposome, in diverse settings, transactionally and longitudinally, with attention to both distal and proximal influences [54] to understand its role in psychopathology/resilience [55]. Setting up longitudinal studies in LMIC, using measures comparable to existing studies can provide a more nuanced understanding of the gene-environment contributions to psychiatric morbidity.

### The focus on externalizing disorders

**Fig. 1:**
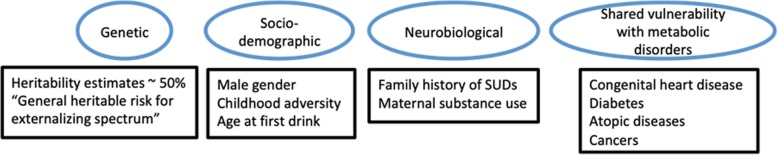
Complex, multi-factorial underpinnings of externalizing disorders [60–66, 79–81]

Externalizing disorders are the third most prevalent class of mental disorders (after anxiety and depression) [56]. These include attention-deficit/hyperactivity disorder (ADHD), Oppositional defiant disorder (ODD), Conduct disorder (CD) in childhood, and adult ADHD, mood disorders, substance use disorders, impulse control disorders, emotionally-unstable personality disorder, and antisocial personality disorder in adulthood. They are associated with significant impairment, and health and non-health sector costs [57, 58]. A dimensional framework of “externalizing psychopathology” encompasses poor impulse control, poor attention allocation, heightened emotional reactivity, verbal and physical aggression, violation of rules, and substance abuse [59]. Individuals at ‘high risk’ for externalizing disorders have different patterns of brain activity, neuroadaptation, cognition, and externalizing temperamental traits [60–66]. A number of studies have documented variations in brain regional volumes [67], regional white matter integrity [68], functional blood flow characteristics [69, 70], social intelligence and corresponding functional brain activations [71], brain activation patterns during response inhibition tasks [72], differences in neurophysiological parameters like P300 and pre-exposure cognitive deficits [62]. Interestingly, network disruptions are incrementally related to externalizing symptoms and are proportional to the alcoholism family density [73]. These variations are also seen as intermediate phenotypes in individuals with externalizing disorders [74–76], suggesting that maturation delays, deficits and deviations predate disorder onset and hold promise as early identification markers of vulnerability. Preliminary investigations have also shown that young adults at ‘high risk’ could ‘catch-up’ on brain maturational differences, emphasizing the role of early interventions [77, 78]. The externalizing spectrum has complex, multi-factorial underpinnings (Fig. 1), strong links with sequential development of various disorders and plausibly a common inherited causality [5]. This makes them particularly interesting in the search for common genetic and neurodevelopmental vulnerabilities and moderating environmental influences.

Key concepts emerging from research on the etiopathological basis of psychiatric morbidity have highlighted – *a genetic and neurodevelopmental continuum, a poly-gene-environmental etiopathogenesis, unique socio-cultural contexts as environmental determinants of psychopathology, variations in brain trajectories leading to different psychopathological outcomes, and the differential impacts of environmental stressors on developmental trajectories over various life-stages*. It follows that studying disparate environmental influences, across the developmental lifespan, on genetically determined trajectories of multimodal brain endophenotypes, using dimensional, multi-modal measures in a longitudinal framework could uncover key etiopathological processes underlying psychiatric morbidity.

### The cVEDA collaboration

The c-VEDA is a collaborative venture between researchers from India and the United Kingdom (UK), set up under a joint initiative on the aetiology and life-course of substance misuse and its relationship with mental illness, by the Medical Research Council, UK (MRC) and the Indian Council for Medical Research (ICMR). National Institute of Mental Health and Neurosciences, Bangalore (NIMHANS) and King’s College London (KCL) are the coordinating centres in India and the UK, respectively. Other participating centres from India include – (i) Post Graduate Institute of Medical Education and Research, Chandigarh (PGIMER), (ii) ICMR-Regional Occupational Health Centre (ROHC), Kolkata, (iii) Regional Institute of Medical Sciences, Imphal (RIMS), (iv) Holdsworth Memorial Hospital, Mysore (HMH), (v) Rishi Valley Rural Health Centre, Chittoor (RV), and vi) St. John’s Research Institute, Bangalore (SJRI). European collaborators include researchers who are part of major longitudinal imaging genetics studies – “Reinforcement-related behaviour in normal development and psychopathology” (IMAGEN) (https://imagen-europe.com), the “Avon Longitudinal Study of Parents and Children” (ALSPAC) (http://www.bristol.ac.uk/alspac/), and the “Study of Cognition, Adolescents and Mobile Phones” (SCAMP) (https://www.scampstudy.org).

### Objectives of the cVEDA study

The cVEDA study is designed to (a) establish a cohort of about 10,000 individuals within specified age bands – 6–11, 12–17, 18–23 years; (b) Conduct detailed phenotypic characterization, with special emphasis on externalizing behaviors (temperament and disorders), in the cohort and parents of all participants; (c) Assess environmental exposures (psychosocial stressors, societal discrimination, nutrition and asset security, environmental toxins) thought to impact gene expression, brain development, temperaments and behaviors; (d) Establish a sustained and accessible data platform and a bio-resource with an integrated database to facilitate analyses and collaborations; and (e) Build research capacity by joint UK–India initiatives.

## Methods/Design

### Study design

**Fig. 2:**
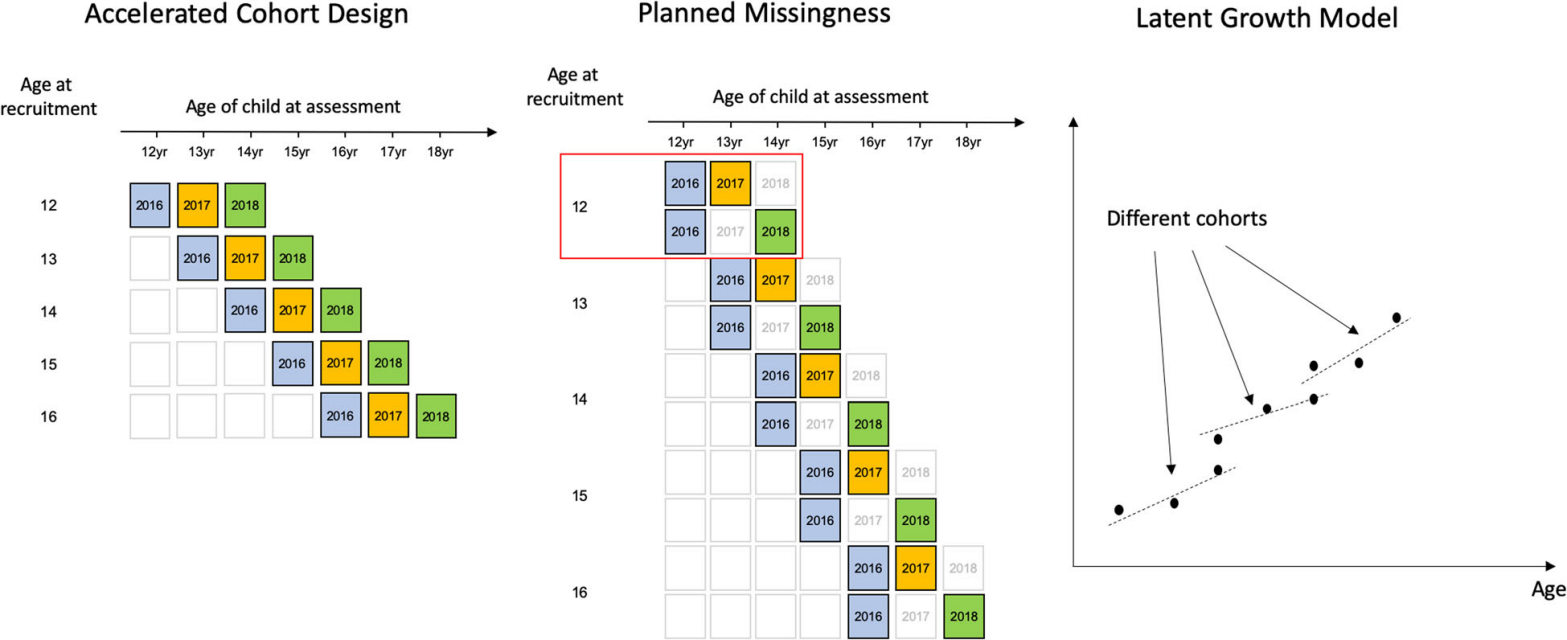
Accelerated longitudinal with planned missingness design and the generation of developmental trajectories (latent growth model)

The cVEDA is a cohort of individuals aged 6–23 years across 7 Indian sites. It draws upon existing research systems with well-established tracking and follow-up mechanisms, previously employed in studying the impacts of varied risk factors on non-communicable diseases [82–85]. Cohorts recruited at each centre are followed-up 1 and 2 years after baseline assessments. The study employs a planned missingness [86] (Fig. 2) approach in which participants are randomised to follow-up either 1 or 2 years after enrolment. Such a design permits three waves of data collection to be achieved while reducing the cost of measurement per wave. Since participants are randomly assigned to be present/missing at each follow-up, missing data are completely at random and hence parameters of interest can be estimated without bias. In addition, loss-to-follow up can be reduced as participants suffer less with study-fatigue and also fieldworkers can target their limited resources more effectively when encouraging participants to return.

Since the primary interest of the study is in tracking development over almost the entire developmental lifespan there are cost/time benefits due to recruiting across a wide range of participant ages. Age-variability within-wave permits an accelerated cohort design [87] to be employed (Fig. 2). Here a given age-range can be spanned in a shorter period of time by considering the cohort as being comprised of multiple sub-cohorts each of a different age at recruitment. A variety of longitudinal statistical models, either within a Structural Equation Modelling or Multilevel Modelling framework, including latent growth models (aka mixed-effects models) can take full advantage of such data. For example, using at joint model one might examine the longitudinal interplay between alcohol use and antisocial behaviour through adolescence. In addition, these models, through their use of a maximum-likelihood approach to missing data, based on a Missing At Random assumption, can demonstrate a high level of statistical power for a fraction of the monetary and time costs of following all individuals for the whole time period. Such an analytical framework is also compatible with the planned-missingness aspect to the study design described above.

### Timeline

**Fig. 3:**
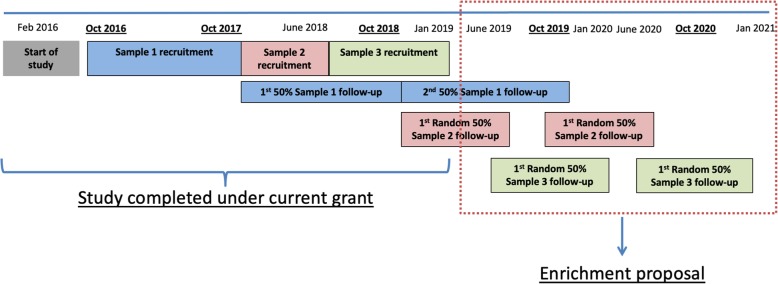
cVEDA study timeline

The cVEDA study started in February 2016. After 9 months spent in study set up (staff recruitment, translations of study instruments into 7 Indian regional languages, setting up digital data capture platform, training of recruitment and assessment teams, and quality control exercises), recruitment started in October 2016. Recruitment, baseline assessments and randomized follow-up have been continued in parallel. Under the current funding cycle, we have completed recruitment and baseline assessments; and 1 and 2 year follow-ups on a part of the sample (Fig. 3).

### Sample

*Baseline Clinical Assessment:* The cVEDA had a target baseline sample size of approximately 10,000 participants in three age bands: C1 (6–11 years), C2 (12–17 years) and C3 (18–23 years). As the objectives of the study were to carry out detailed phenotypic characterisation of participants, examine their environmental exposures and gene-environment interactions that may modulate brain development and affect externalizing and addictive behaviour patterns, the study sample needed to be as large as feasibly possible whilst being representative of the geographic, socioeconomic and cultural diversity of India. The target sample sizes at each recruitment site were decided based upon each site’s estimated capacity to recruit individuals over a 3-year recruitment period, given the site lead’s understanding about ground realities and experience from past studies. The sample represents five geographically, ethnically, and socio-culturally distinct regions; varied environmental risks: toxic exposures (coal-mines), slum-dwellers, socio-political conflict zones (insurgency and inter-ethnic violence); urban and rural areas; school and college attendees; and familial high risk (children of parents with substance use or other mental disorders), in order to have an adequate representation of individuals likely to convert to externalizing disorders.

**Fig. 4:**
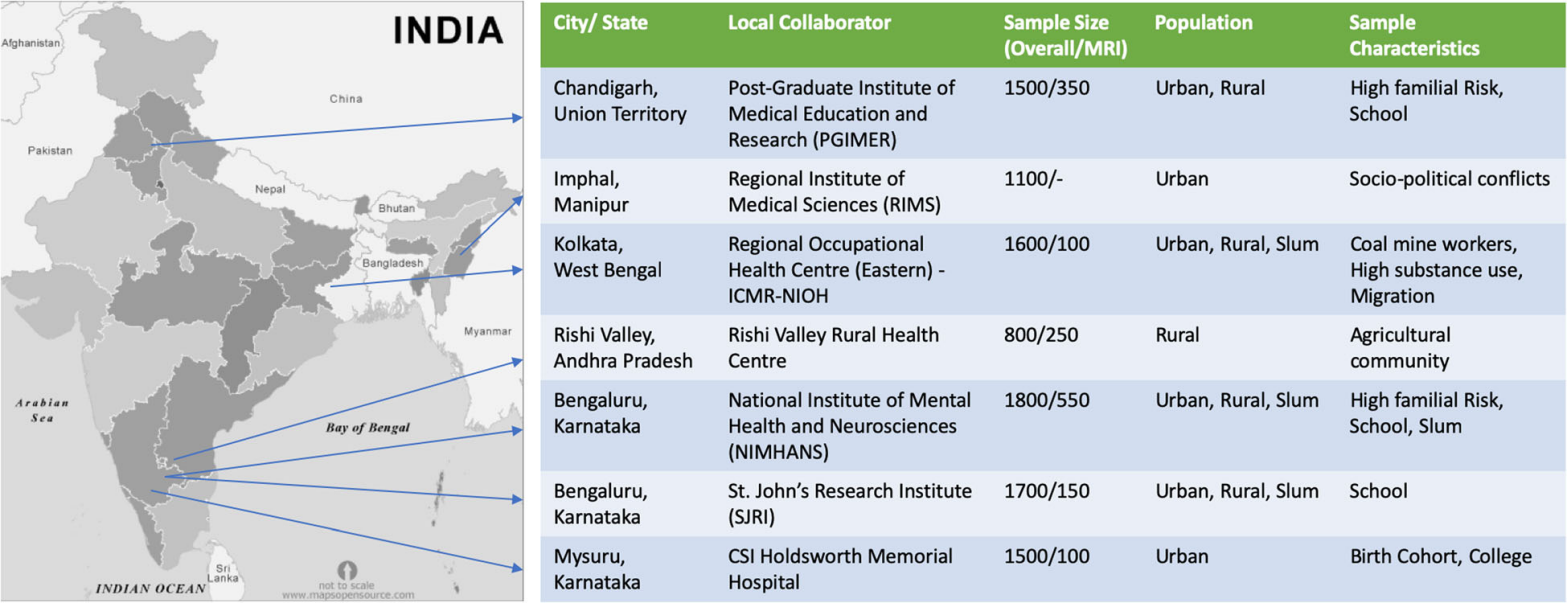
cVEDA sample distribution and recruitment site characteristics (Map of India source: http://mapsopensource.com/india-states-outline-map-black-and-white.html; As stated on the webpage “All the content by www.mapsopensource.com is licensed under a Creative Commons Attribution 3.0 Unported License”)

The study followed non-probabilistic convenience sampling based on accessibility to potential participants in local schools, colleges, community and clinics. Exclusion criteria included legal blindness/deafness, seizure disorder active in the last 1 month, severe physical or active mental illness, refusal of consent, or inability to participate in follow-up assessments (e.g. due to migration). Individuals with specific contra-indications (metal implants, electrical devices, severe claustrophobia) were excluded from neuroimaging. Site-wise exposure characteristics and baseline sample sizes are depicted in Fig. 4. Whilst the recruitment approach was pragmatic, this type of sampling technique may not allow for results that can be generalised to the entire population. However, given the scope and breadth of exposures being assessed, this approach is useful for the target research objectives and may help generate new hypotheses for future studies [88].

*Biological samples and neuroimaging:* Blood/buccal swab and urine samples were collected from participants at baseline. Around 15% of the baseline sample, i.e. consenting participants, underwent neuroimaging. Even at the risk of biased sampling, this strategy was adopted given the wide age range, high rate of refusal, especially in non-clinical populations, and the unavailability of research MRI scanners in 4 out of 7 sites.

## Procedure

### a) Phenotypic characterization

Assessments involved dimensional and categorical phenotypic characterization. The questionnaires and assessment protocols were translated (and back-translated using standard WHO protocols) from English into seven Indian languages (Hindi, Kannada, Telugu, Tamil, Manipuri, Bengali, Punjabi) for use across seven recruitment sites. Age-appropriate instruments are used to capture socio-demographic information, temperament, environmental exposures, parenting, psychiatric morbidity, and neuropsychological functioning. Table 2 details the domains of assessments, tools and protocols.

**Table 2:** cVEDA sample characterisation: Assessment domains, tools& protocols

Assessment domain	Questionnaires	6–11 years	12–17 years	18–23 years	Follow-up
Socio-demographic information	Socio-demographic questionnaire [89]	**✓**	**✓**	**✓**	**✓**
	Migration questionnaire [90]	**✓**	**✓**	**✓**	**✓**
Exposures questionnaires	Environmental exposures questionnaire [91]	**✓**	**✓**	**✓**	✓
	Adverse childhoodexperiences – International questionnaire [37]	✓	✓	✓	
	Children’s Revised Impact of Event Scale [92]				
	Short food questionnaire *(modified Food Frequency Questionnaire)* [93]	✓	✓	✓	
	Pregnancy History Instrument – Revised [94]	✓	✓	✓	
	Indian Family Violence and Control Scale [95]	✓	✓	✓	
	Mobile use questionnaire (Self-report) [96]		✓	✓	
	Mobile use questionnaire (Parent-report) –*from the SCAMP study* (https://www.scampstudy.org)		✓		
	Life Events Questionnaire [97]			✓	
	Questions on urbanicity (*devised to explore all places a participant has successively resided at)*	✓	✓	✓	
Parenting	Alabama parenting questionnaire – Child & Parent [98, 99]	✓	✓		
	Adolescent attachment questionnaire [100]		✓		
	Parental bonding instrument [101]			✓	
Temperament	Childhoodbehavior questionnaire [102]	✓			
	Early adolescent temperament questionnaire [103]		✓		
	Adult temperament questionnaire [104]			✓	
	Big Five Personality inventory [105]			✓	
	Strengths & difficulties questionnaire – Parent [106]	✓	✓		✓
	Strengths & difficulties questionnaire – Child [107]		✓		✓
	Strengths & difficulties questionnaire – Self-report [107]			✓	✓
Psychiatric morbidity	MINI-KID [108]	✓	✓		✓
	MINI-5 [109]			✓	✓
	ASSIST-Plus [110–113]	✓	✓	✓	✓
	ASRS – ADHD [114]			✓	
Family history	Family history questionnaire *(clinical assessment for presence of medical and/or psychiatric disorders in first-degree relatives of the participant)*	✓	✓	✓	
Medical history	Medical problems questionnaire *(clinical assessment for presence of medical disorders in the participant)*	✓	✓	✓	
Puberty	PubertalDevelopmentScale [115]	✓	✓		
Neuropsychological assessment	Psychology Experiment Building Language (PEBL)[96]	✓	✓	✓	✓
	Digit span test – forward and reverse				
	Corsi block test – forward and reverse				
	Now or later test				
	Trail making test				
	Sort the cards				
	Stop signal task [122]				
	Balloon analogue risk task [121]				
	Emotion recognition task				
	Social Cognition Rating Tool in the Indian Setting [116]				
Anthropometry	Height	✓	✓	✓	✓
	Weight				
	Mid arm circumference				
	Leg length				
	Head circumference				
Neuroimaging	Structural MRI	✓	✓	✓	✓
	T1-weighted, 3D magnetization prepared gradient echo sequence (MPRAGE) based on the ADNI protocol (http://www.loni.ucla.edu/ADNI/Cores/index.shtml):				
	T2 weighted fast- (turbo-) spin echo				
	FLAIR scans				
	Diffusion MRI				
	Single-shot spin-echo EPI sequence				
	Single acquisition session				
	Acquisition repeated with reversed blips				
	Resting state functional MRI BOLD functional images acquired with a gradient- echoplanar imaging (EPI) sequence, using a relatively short echo-time to optimize reliable imaging of subcortical areas.				
Toxicology	Urinary volatile organic compounds	✓	✓	✓	
	Solid phase extraction followed by High Performance Liquid Chromatography.				
	Urinary Arsenic				
	Flow injection system by Atomic				
	Absorption Spectrometer (PerkinElmer AA800, USA)				
	Plasma lead				
	Transversely-heated graphite furnace and Zeeman background correctionusing Graphite Furnace Atomic Absorption Spectrometer (PerkinElmer AA800, USA)				

### Neuroimaging

Resting state fMRI (rsfMRI), Diffusion MRI (dMRI) and Structural MRI (sMRI) scans are done at baseline, and are being done for the randomized consenting participants at follow-up. Structural and rsfMRI are collected on 3 T scanners (Siemens, Germany; Philips, The Netherlands). To ensure comparability of image-acquisition techniques and ‘pool’ability of the multi-site MRI data, a set of parameters, particularly those directly affecting image contrast or signal-to-noise are held constant across sites (https://cveda.org/standard-operating-procedures/) (Table 2).

### Blood/saliva samples for genetic studies

*Blood samples* (at least 10 ml, EDTA and Tempus tubes) are collected at baseline for DNA, RNA and plasma lead estimation, as per a Standard Operating Protocol (https://cveda.org/standard-operating-procedures/). Plasma, ‘buffy coat’ (white blood cells) and red blood cells, from centrifugation of blood samples in EDTA tubes, are transferred into labeled aliquots for storage at a central biobank. Tempus tubes and blood component aliquots are stored at − 80 °C. Samples are kept frozen at all times including during transport using temperature-controlled logistics. For participants who do not consent for a blood sample, or where it isn’t possible to obtain a blood sample (e.g. failure to identify a suitable vein for blood draw or an insufficient amount of blood sample), a *buccal swab* (for DNA) is taken.

### Plasma and urine samples for toxicological studies

Estimation of lead in plasma, and arsenic, metabolites of tobacco (cotinine) and volatile organic compounds (VOCs), in urine samples, is incorporated in cVEDA as a measure of exposure to environmental neurotoxins. An aliquot of plasma isolated from blood samples is used for lead estimation. Lead is estimated in plasma as evidence suggest that plasma lead represents the toxicologically labile fraction of lead freely available to interact with target tissue rather than lead in whole blood [117, 118]. Mid-stream *urine* samples collected in sterilized and capped polythene bottles, and stored in deep freezers at −20 °C till analysis. Urine samples are analysed for total arsenic and metabolites of VOCs include trans, trans-muconic acid and s-phenyl mercapturic acid (benzene metabolites), hippuric acid (toluene metabolite), mandelic acid (ethylbenzene metabolite) and methylhippuric acid (xylene metabolite). Analytical methods for toxicological analysis are presented in Table 2. To validate the toxicological assessments in participants, environmental assessments of VOCs in ambient air and arsenic in water will also be carried out. In a phased manner, exposure to other critical developmental neurotoxins, like pesticides, phthalates and polychlorinated biphenyls, will also be assessed.

### Follow-up assessments

At follow-up, assessments relevant to tracking development, changes in environmental exposures, and psychopathology are done. These include – socio-demographic and migration information, environmental exposures, MINI/MINI-KID, Strengths and Difficulties Questionnaire, ASSIST-Plus, and neuroimaging. MRI scans are repeated for those participants who underwent neuroimaging at baseline and consented for a repeat scan in follow-up. These would help track structural and functional brain growth trajectories. Blood/buccal swab samples are also collected from all consenting participants in follow-up, for epigenetic investigations.

### Data analysis

#### Handling missing data

Attrition, difficulties in assessment within a set follow-up time frame, changes in recruitment techniques (face-to-face interviews to telephonic-based assessments), changes in reporting individuals (mother to child or vice versa), and, changes in questionnaire design are challenges in longitudinal cohorts. In cVEDA, we also have missing data from exogenous factors such as economic migration, weather catastrophes and political instability resulting in displacement of participants from one geographical area to another. These would be significant caveats to fitting longitudinal growth models for behaviour problems. “Planned missingness” improves the efficiency of longitudinal studies without compromising validity. As this missingness is by design we are able to assume that data are ‘missing completely at random’ and hence likelihood-based methods would produce unbiased effect estimates. Planned missingness can also affect the level of unplanned missingness. By reducing the number of waves of data that each participant contributes, participant-fatigue is reduced, study-dropouts are lower and, thereby, the rates of unplanned missingness.

### Statistical analysis and modelling

The cVEDA will have first and foremost a descriptive analysis, as it is the first neurodevelopmental study of its kind in India with a diverse socioeconomic, geographic and cultural spread. It will present findings on key externalizing traits across the cohort with cluster analysis conducted per site to determine effects attributable to site variations. The results of cVEDA will be informed by and compared with the findings of European cohorts of children and young adults such as IMAGEN, ALSPAC, and SCAMP for behavioural traits (temperament and disorders), environmental exposures (e.g. psychosocial stressors and environmental toxins) and gene-environment interactions. Further, techniques like structural equation models [119] that estimate and converge multiple pieces of the outcome into a single latent growth or specific latent classes by age will be used to identify vulnerability factors for externalizing disorders and other mental health outcomes.

### Establishment of a repository data and biobank

All cVEDA assessments are run on a digital platform using *Psytools software* (Delosis Ltd., UK). Data is first synchronized with the Psytools server, from where it is accessed by data management teams in India, UK and France. The final data storage server (Dell power edge R530 Rack server) is located at NIMHANS, along with a back-up safety system and a mirror data storage system in France. The data management team conducts sanity checks and feedback is regularly sent to recruitment sites.

Biological samples form part of a biobank located at NIMHANS. The databank and the biobank are a resource to facilitate research by consortium partners as well as collaborators investigating other areas of mental health, its interface with physical health, and cross-cultural comparisons.

### Quality control

Quality control measures have been incorporated in the study protocol right from the beginning. Interviewer training, on-site and online, followed by mock interview assessments and feedback was conducted in the preparatory phase of the study. Following start of study on the field, India and UK based study coordinators conduct weekly recruitment meetings during which recruitment progress and completeness of data entry are reviewed. Standard operating procedures (SOPs) ensure consistency in biological samples collection and neuroimaging. Additionally, quality control procedures for neuroimaging are implemented at each site: (i) a phantom [120] is scanned to provide information about geometric distortions and signal uniformity related to hardware differences in radiofrequency coils and gradient systems, (ii) healthy volunteers are regularly scanned at each site to assess factors that cannot be measured using phantoms alone, and iii) after every subject scan, a quick 2-min script (https://github.com/cveda/cveda_mri) is run at the acquisition centre to detect any subject−/scanner-related artifacts and a decision is made if the data needs to be re-acquired. The India-based study coordinator also regular conducts site visits to monitor adherence to the study protocol.

## Discussion

This paper presents the background and protocol of the cVEDA study. The study is designed to answer questions about the developmental, genomic, and environmental underpinnings of psychopathology. This has implications for preventive and early interventions for mental disorders. Biological samples collected from all participants significantly enrich exposome characterization in the sample and this will aid in discovering biomarkers of exposure and early disease through omic technologies (epigenomics, adductomics, proteomics, transcriptomics and metabolomics). Whilst it is beyond the scope of the current funding to carry out extensive omic analyses, we are establishing an integrated exposome database and biobank to facilitate future analyses. The cVEDA cohort is a substantial addition to, and provides comparative and cross-cultural datasets for international studies – IMAGEN that looks at biological and environmental factors affecting reinforcement related behaviours in teenagers; SCAMP looking at effects of mobile phone use on cognition in adolescents; ALSPAC with several decades of longitudinal data on developmental, environmental and genetic factors affecting a person’s overall health and development.

Our initial analyses have focused on establishing trajectories (behavioural, temperamental, neuropsychological) to increase our understanding of maturational brain changes and permit in-depth enquiries into the relationships between brain networks, cognition, behavior and environment during development, and how these contribute to the genesis of neurodevelopmental disorders.

Genomic DNA isolated from the buffy coat component of blood will be used for genetic studies, using next generation genotyping methods as well as genome wide epigenetic studies. RNA from samples stored in Tempus tubes will be used for transcriptome studies by arrays or RNA sequencing methods. Using statistical data reduction techniques (e.g: principal components analysis), endophenotype-genetics relationships (e.g: parallel independent component analysis), and quantitative trait loci, we will examine genetic basis of neuroimaging and neuropsychological endophenotypes.

### Community engagement and capacity building

In addition to being a research initiative, cVEDA is an opportunity for community engagement. At the completion of the study we will provide a summary note to participants and an opportunity for group discussion of results, implications and treatment options, where relevant. The cVEDA consortium facilitates exchange of research, technical and statistical expertise and support with dissemination and publication of research findings via workshops and training programmes organised annually at the cVEDA investigators’ meetings at various study sites in India.

cVEDA is a first of its magnitude research exercise in India, an opportunity for research growth and capacity building. The establishment of a completely digitized data collection and transfer platform minimizes human error greatly. It also makes a large databank rapidly available to researchers working in the field. Through collaboration with institutions in the UK, research scholars also have the opportunity to partner with international researchers and faculty to build their research repertoire.

In conclusion, the cVEDA has established the largest neurodevelopmental database in India. The identification of environmental risk factors that contribute to vulnerabilities for psychiatric morbidity could have huge implications in public health interventions and prevention of psychiatric morbidity, in India. This unique database will facilitate international research collaborations, to study cross-cultural variations in the determinants of psychopathology.

## Data Availability

The dataset generated during the cVEDA study are available to interested researchers as per the cVEDA data sharing guidelines (https://cveda.org/access-dataset/).

## Abbreviations

ADHD: Attention Deficit Hyperactivity Disorder
ALSPAC: Avon Longitudinal Study of Parents and Children
ASSIST: Alcohol, Smoking and Substance Involvement Screening Test Consortium on
CD: Conduct Disorder
cVEDA: Vulnerability to Externalizing Disorders and Addictions
dMRI: Diffusion Magnetic Resonance Imaging
fMRI: Functional Magnetic Resonance Imaging
ICMR: Indian Council of Medical Research
IMAGEN: The IMAGEN study: reinforcement-related behaviour in normal brain function and psychopathology
KCL: King’s College London
LMIC: Low and Middle Income Countries
MINI: Mini International Neuropsychiatric Interview
MINI-KID: Mini International Neuropsychiatric Interview for Kids
MRC UK: Medical Research Council, UK
MRI: Magnetic Resonance Imaging
NIMHANS: National Institute of Mental Health and Neuro Sciences
ODD: Oppositional Defiant Disorder
rsfMRI: Resting state functional Magnetic Resonance Imaging
SCAMP: Study of Cognition Adolescents and Mobile Phones
sMRI: Structural Magnetic Resonance Imaging
SOPs: Standard Operating Procedures
VOCs: Volatile Organic Compounds
